# Parental Obesity and Offspring Risk for Cardiorenal Diseases

**DOI:** 10.1007/s11906-026-01379-2

**Published:** 2026-08-20

**Authors:** Jussara M. do Carmo, John E. Hall, Zhen Wang, Alan J. Mouton, Ana C. M. Omoto, Luciana Jorge, Alexandre A. da Silva

**Affiliations:** Department of Physiology and Biophysics, Mississippi Center for Obesity Research, Cardiorenal and Metabolic Diseases Research Center, University of Mississippi Medical Center, 2500 North State Street, Jackson, MS USA

**Keywords:** Development programming, Cardiovascular diseases, Chronic kidney disease, Acute kidney injury, Myocardial infarction, Heart failure, Metabolic syndrome

## Abstract

**Purpose of Review:**

Obesity rates have increased dramatically during the past four decades, and these increases have occurred in children and adolescents, as well as in adults at reproductive age. Currently, 40.3% of men and 39.7% of women 20–39 years of age in the United States are classified as obese, leading to a substantial rise in parental obesity. Obesity is a major risk factor for cardiovascular, metabolic and renal diseases, including hypertension, type 2 diabetes, and chronic kidney diseases. This review examines current evidence of how parental obesity programs offspring susceptibility to cardiorenal and metabolic dysfunction and discusses the potential underlying mechanisms that contribute to the long-term health consequences in the offspring.

**Recent Findings:**

Increasing evidence indicates that parental obesity, particularly maternal obesity, predisposes offspring to cardiovascular, metabolic, and renal dysfunction that can emerge early in life and persist into adulthood, even when controlled for lifestyle factors. Although alterations in the intrauterine and early postnatal environments are believed to underlie developmental programming, the mechanisms by which parental obesity influences long-term offspring health remain poorly understood. Emerging evidence suggests that dysregulation of neurohumoral pathways, chronic inflammation, mitochondrial dysfunction, and intercellular signaling may be transmitted across generations and contribute to increased risk of cardiorenal and metabolic diseases.

**Summary:**

Elucidating these mechanisms is essential for identifying novel therapeutic targets and determining whether interventions implemented before conception, during pregnancy, or during early life can mitigate adverse health outcomes and improve long-term offspring health.

## Introduction

The prevalence of overweight and obesity among women and men of reproductive age has steadily increased in the past four decades, with no signs of decline. More than 20 percent of children and adolescents in the U.S. are currently obese (CDC Obesity) [1]. The developmental origins of health and disease concept proposes that non-communicable diseases, including cardiovascular disease (CVD), may originate from adverse environmental exposures during preconception, in utero, and early infancy [2–4]. While the majority of observational and experimental studies have focused on parental programming of metabolic diseases, far fewer have examined cardiorenal dysfunction, particularly when both parents are obese at the time of conception.

**Fig. 1:**
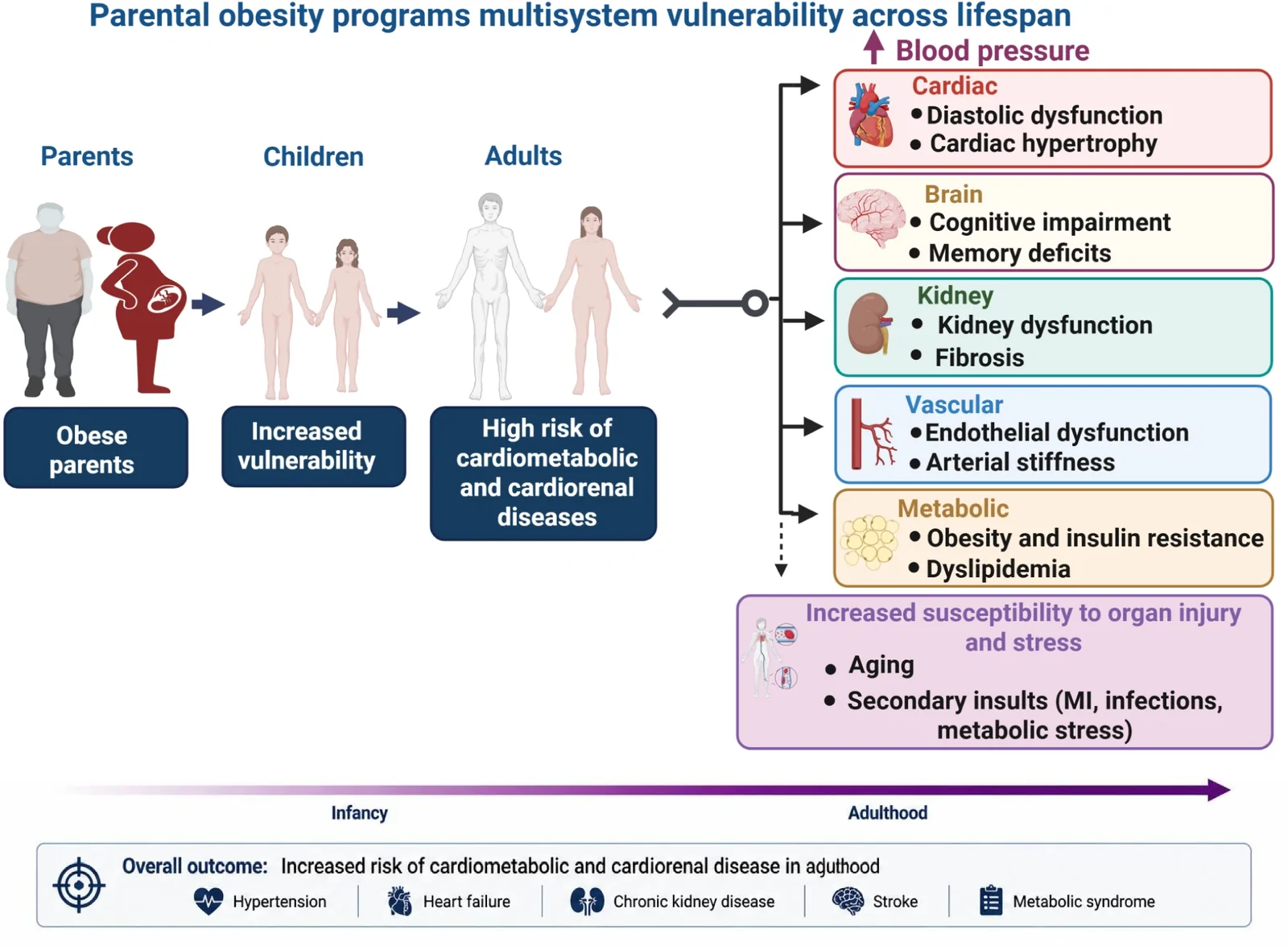
Parental obesity and offspring susceptibility to cardiorenal diseases. Parental obesity programs offspring to early cardiovascular dysfunction that progresses with aging

**Fig. 2:**
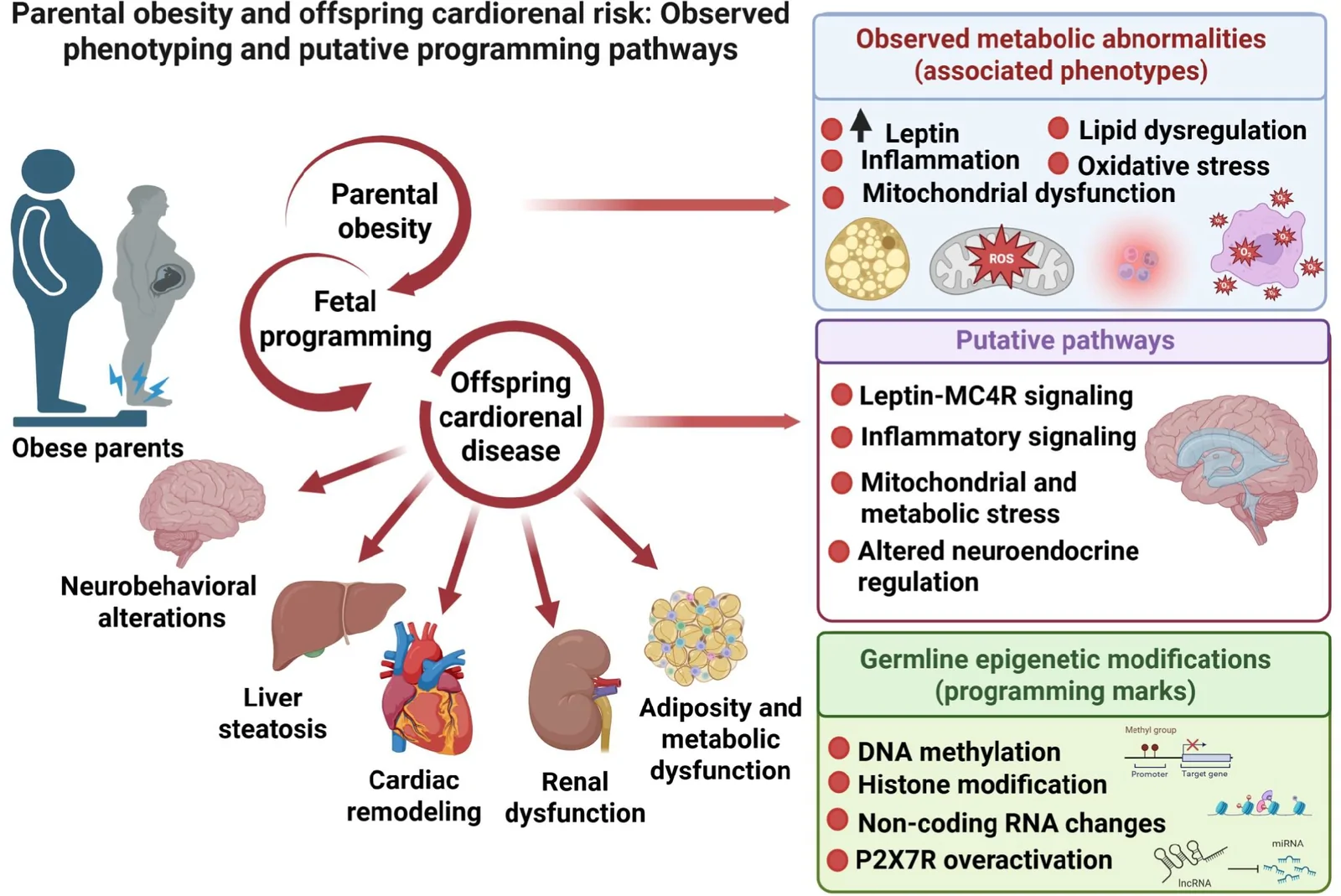
Mediators of parental obesity-induces cardiorenal diseases

As the number of children born from parents with high adiposity continues to rise globally, understanding the intricate interplay among genetic, developmental, and environmental factors that influence offspring health has become increasingly critical. Given the strong association between parental obesity and increased susceptibility to cardiovascular, metabolic and renal diseases in offspring, this review summarizes current evidence on the impact of maternal and paternal obesity on offspring cardiovascular and renal outcomes (Fig. 1). We highlight the pathophysiological alterations associated with parental obesity and discuss how these changes may predispose offspring to hypertension, cardiac dysfunction, chronic kidney disease (CKD), and cardiorenal injury throughout the lifespan (Fig. 2).

## Adverse Effects of Parental Obesity on Offspring Cardiac and Renal Function Later in Life

As the prevalence of overweight and obesity continues to rise worldwide, an increasing number of offspring will be born from obese parents, increasing the risk of obesity and cardiorenal diseases in future generations. Preconception and antenatal lifestyle modifications or other forms of anti-obesity therapies in parents could play a crucial role in preventing adverse cardiovascular outcomes in their offspring.

### Parental Obesity and Offspring Cardiorenal Health

Epidemiological studies indicate that children from obese parents have 10–12 times greater probability of developing obesity and metabolic dysfunction compared to children of parents with normal body weight [5, 6]. Thus, having overweight or obese parents significantly increases the risk of obesity in childhood and adulthood [7, 8]. Beyond increased adiposity, offspring of obese parents frequently show a cluster of metabolic disorders, including insulin resistance, glucose intolerance, dyslipidemia, and altered fatty acid metabolism, even before the onset of obesity [5, 9]. These metabolic alterations are often accompanied by low-grade systemic inflammation, increased oxidative stress, and impaired mitochondrial function, which together predispose the offspring to endothelial dysfunction, hypertension, and cardiorenal diseases [9, 10] (Fig. 1).

Experimental studies from animal models further support these findings. Several studies indicate that parental obesity programs adverse renal function in offspring. For example, offspring from obese mothers are more susceptible to renal damage following streptozotocin-induced diabetes [11, 12]. Maternal obesity is linked to impaired nephrogenesis, glomerular hypertrophy, increased lipid accumulation, and early activation of profibrotic and inflammatory pathways [13–17]. These findings suggest that parental obesity not only programs susceptibility to obesity and metabolic syndrome, but also establishes a pro-inflammatory, pro-oxidative, and lipotoxic milieu that impairs cardiovascular and renal health across generations (Fig. 2). We demonstrated that male and female offspring from obese parents exhibit higher kidney injury scores, with male offspring showing greater vulnerability to the detrimental effects of parental obesity [18]. In fact, even when they were fed a normal diet and maintained a lean phenotype with no prior insults, male offspring from obese parents exhibited increased inflammatory cell infiltration and more pronounced renal damage at a young age (22 weeks old) compared to age-matched male offspring from lean parents [18]. Furthermore, after an acute kidney insult (i.e., ischemia–reperfusion injury), male offspring from obese parents exhibited more severe renal damage compared to male offspring from lean parents and their female littermates [18]. These findings highlight significant programming effects of parental obesity on their offspring’s susceptibility to renal injury that can occur independently of obesity but are influenced by the sex of the offspring.

Mechanistically, similar cellular pathways that contribute to renal injury also underlie early cardiac dysfunction in the offspring of obese parents. We previously demonstrated that parental obesity programs offspring to develop cardiac dysfunction as early as 21–23 days of age, which was associated with reduced cardiac mitochondrial function and expression of Sirtuin 3 (SIRT3), a critical regulator of mitochondrial function, in male rats [19]. Additionally, we found that male and female offspring from obese parents exhibited increased blood glucose, plasma insulin, and leptin concentrations as early as 21–23 days after weaning. We also observed early signs of cardiac diastolic dysfunction, evidenced by increased tau, end-diastolic pressure–volume relationship (EDPVR), isovolumetric relaxation time (IVRT), and E/e′ ratio, along with reduced E/A values in young male, but not female, offspring from obese parents [19]. These findings from a rodent model of parental obesity suggest important sex differences in the impact of parental obesity on early-onset cardiac dysfunction, with females exhibiting greater resistance to the adverse influences of parental obesity compared to their male siblings at three weeks of age.

Parental obesity also programs their offspring to develop elevated blood pressure and exaggerated blood pressure responses to acute stress. For example, we found that male offspring from obese parents exhibited increased blood pressure and heightened blood pressure responses to acute air-jet stress, independent of the diet consumed after weaning. In contrast, female offspring showed no alterations in baseline blood pressure or stress-induced blood pressure responses, despite having similar adiposity as their male siblings [20]. These findings suggest that male offspring are more susceptible to the hypertensive effects of parental obesity.

## Potential Mechanisms of Cardiorenal Dysfunction in Offspring of Obese Parents

Multiple mechanisms may be involved in mediating the programming of cardiorenal diseases in offspring from obese parents. Here, we highlight potential major factors that may explain the increased susceptibility to cardiovascular and renal dysfunction in offspring from obese parents.

### Sympathetic Nervous System Overactivation

It is important to clearly distinguish between the direct pathophysiological consequences of obesity and the mechanisms by which parental obesity may influence offspring health. In individuals with obesity, activation of the sympathetic nervous system (SNS) is a key factor contributing to a range of obesity-related health issues, including hypertension and CVD**.** The SNS plays a central role in regulating cardiovascular, renal and metabolic function and its overactivation in obesity is associated with adverse physiological outcomes [21–24]. For instance, obesity is associated with renal SNS activation, which increases renal sodium retention leading to volume expansion and increased blood pressure which, in turn, is a major risk factor for CKD [16, 25].

Our previous findings demonstrated that increased sympathetic activation contributes to elevated blood pressure in offspring from obese parents [20], even when the offspring are fed a normal diet and remain lean. Specifically, we observed that ganglionic blockade with hexamethonium resulted in a greater reduction in blood pressure in lean and obese offspring from obese parents, compared to lean and obese offspring from lean parents [20].

Our studies also demonstrated that male offspring from obese parents exhibit increased 24-h blood pressure lability and blood pressure responses to acute stress, independent of the diet consumed by the offspring after weaning [20]. Notably, this response was not observed in female offspring, suggesting that sex hormones, genetic factors or others factors may confer protection to female offspring against the adverse effects of parental obesity on blood pressure regulation, at least during young adulthood. Additionally, we observed a higher low frequency (LF) component of systolic arterial pressure oscillation, indicative of increased sympathetic tone in obese male offspring from obese parents [20]. This finding supports the hypothesis that increased sympathetic activity contributes to greater blood pressure lability in these offspring. However, the long-term implications of these findings for increased CVD risk in offspring of obese parents as they age will require further investigation. Also, whether the sex differences observed at young ages in offspring from obese parents disappear or are exacerbated with aging remains to be determined.

### Leptin-Melanocortin System

The leptin-melanocortin system plays an important role in regulating energy balance, body weight, and various aspects of metabolic function. Increased levels of leptin, a hormone produced by adipocytes, inform the brain to reduce appetite and increase energy expenditure [26–29]. The melanocortin system, which includes melanocortin receptors (particularly MC4R), is involved in these processes. The interaction between leptin and melanocortin receptors helps maintain the body’s energy balance as well as influencing blood pressure.

Parental obesity, particularly maternal obesity, programs offspring for cardiometabolic abnormalities. Maternal obesity may disrupt the normal development of leptin dependent neural circuits in offspring, leading to long-term dysfunction in energy balance and cardiovascular regulation. Changes in leptin levels or sensitivity during critical developmental windows may impair leptin sensitivity and alter melanocortin system function, thereby predisposing offspring to obesity, insulin resistance, and hypertension later in life, affecting the regulation of appetite, energy expenditure, and fat storage [30, 31] (Fig. 2).

Previous studies suggest that offspring of obese mothers may have increased activation of melanocortin signaling, which could contribute to altered metabolic responses and increased susceptibility to hypertension [30, 32–34]. For instance, Samuelsson and colleagues [33] demonstrated that offspring from obese mothers develop hyperleptinemia, elevated blood pressure, and renal dysfunction in wild-type, but not in melanocortin-4 receptor (MC4R) knockout (KO) mice. Their data suggest that increased leptin can induce overactivation of MC4R in the paraventricular nucleus of the hypothalamus (PVN) which appears to be required for programming hypertension in offspring of obese parents [33].

We previously demonstrated that the brain melanocortin system plays a key role in parental obesity-related hypertension, potentially through increased sympathetic activity [34]. Pharmacological blockade of MC4R produced a greater reduction in blood pressure in obese male offspring from obese parents, suggesting that increased MC4R activation contributes to elevated sympathetic activity and hypertension in this model [34]. Notably, these effects were more pronounced in males than females, indicating that parental obesity may differentially influence sympathetic regulation in a sex-dependent manner. Several important questions remain unresolved. First, the specific neural circuits mediating MC4R-dependent increases in blood pressure have not been identified. Future studies should determine whether key autonomic regulatory regions, including the PVN and the rostral ventrolateral medulla (RVLM), contribute to enhanced sympathetic outflow in offspring from obese parents. Second, because MC4R activation modulates sympathetic nervous system activity through multiple intracellular signaling pathways that remain incompletely understood, additional studies are needed to identify the downstream pathways responsible for the exaggerated sympathetic activation and hypertension in offspring from obese parents. Third, direct and indirect assessments of sympathetic nervous system activity, including renal sympathetic nerve activity, norepinephrine turnover, and heart rate variability could help establish whether sympathetic tone is elevated in this model and whether such changes are associated with enhanced MC4R signaling. Finally, future studies should determine whether sex hormones contribute to the observed sex differences and whether alterations in leptin sensitivity influence MC4R-dependent regulation of sympathetic activity and blood pressure regulation in offspring from obese parents.

### P2X7R Overactivation and Inflammation

Purinergic receptor 7 (P2X7R) is a key ATP-gated ion channel involved in inflammatory responses, and its overactivation is implicated in various pathophysiological conditions, including hypertension and renal damage [35–38]. We demonstrated that male offspring from obese parents exhibit increased P2X7R protein expression in the kidneys, and that P2X7RKO mice are protected from parental obesity-induced hypertension and renal injury [20].

Parental obesity, particularly maternal obesity, is known to induce chronic low-grade inflammation in their offspring [9, 39], potentially priming the immune system toward a heightened pro-inflammatory state. Metabolic dysfunction and cellular stress can increase extracellular ATP release, leading to sustained activation of the ATP-gated P2X7R. P2X7R stimulation promotes NLRP3 inflammasome assembly and activation, resulting in caspase-1-dependent maturation and release of the pro-inflammatory cytokines IL-1β and IL-18. This pathway has emerged as a key mechanism linking metabolic stress to chronic inflammation and tissue injury [40–43].

Chronic inflammation may contribute to elevated blood pressure through its effects on endothelial function, vascular remodeling, oxidative stress, and renal sodium handling. Consistent with this concept, we recently demonstrated that parental obesity increases offspring blood pressure and their cardiovascular response to stress in offspring in a sex-dependent manner, with male offspring exhibiting a greater hypertensive phenotype than females [20]. Moreover, genetic deletion of P2X7 receptors (P2X7R) attenuated the increase in blood pressure and reduced blood pressure lability in offspring from obese parents, supporting a role for P2X7R signaling in parental obesity-induced hypertension [20]. P2X7R signaling has emerged as an important mediator of renal inflammation and injury [41, 42]. In Dahl salt-sensitive rats, pharmacological inhibition of P2X7R reduced hypertension and renal injury, while studies in angiotensin II-induced hypertension demonstrated that P2X7R blockade decreases renal inflammation and improves renal function [44]. Furthermore, we recently reported that parental obesity predisposes offspring to kidney dysfunction and increases susceptibility to acute kidney injury, particularly in male offspring, suggesting that inflammatory pathways may contribute to the cardiovascular and renal consequences of parental obesity [18], and that genetic deletion of P2X7 receptors (P2X7R7) attenuated the increase in blood pressure and reduced blood pressure lability in offspring from obese parents [20].

Collectively, these evidences suggest that P2X7R signaling may contribute to hypertension and renal injury in offspring from obese parents. However, the relative contributions of inflammation, autonomic dysfunction, endothelial impairment, and renal injury to these outcomes remain poorly defined. Future studies addressing these pathways may provide important mechanistic insights and identify novel therapeutic strategies to reduce the long-term cardiorenal consequences of parental obesity.

### DNA Methylation

DNA methylation, the addition of a methyl group to cytosine residues within CpG dinucleotides, is a key epigenetic mechanism that regulates gene expression, and is implicated in developmental programming of cardiovascular, metabolic, and renal diseases [45, 46]. Evidence suggests that parental obesity induces alterations in DNA methylation patterns in germ cells, placental tissue, and offspring organs, leading to persistent changes in gene expression that may influence disease susceptibility later in life. For instance, obesity alters DNA methylation at imprinted genes in human sperm [47], and obese men exhibit distinct sperm DNA methylation profiles that are partially remodeled by bariatric surgery [48]. Also, offspring from obese parents showed altered methylation patterns at multiple imprinted loci, supporting the role for parental obesity in epigenetic reprogramming across generations [49].

Maternal and paternal obesity are associated with altered methylation of genes involved in energy metabolism, insulin signaling, inflammation, and cardiovascular regulation [47, 48]. Thus, epigenetic modifications may influence pathways involved in adiposity, glucose homeostasis, blood pressure regulation, and renal function, thereby increasing offspring susceptibility to obesity, insulin resistance, hypertension, and CKD. Consistent with this concept, parental obesity has been shown to program hypertension, autonomic dysfunction, and increased susceptibility to renal injury in offspring [18, 20], although the contribution of DNA methylation to these phenotypes remains incompletely understood.

Despite growing evidence linking parental obesity to epigenetic reprogramming, the specific DNA methylation signatures that contribute to cardiorenal dysfunction remain poorly defined. Future studies are needed to identify the genes and pathways most affected by parental obesity and to determine whether these epigenetic changes are causally involved in disease development. Therapeutic approaches targeting DNA methylation, including nutritional interventions that influence one-carbon metabolism and DNA methyltransferase (DNMT) activity, may represent potential strategies to reduce the long-term cardiorenal consequences of parental obesity.

### Extracellular Vesicles

Extracellular vesicles (EVs), including exosomes, microvesicles, and apoptotic bodies, are important mediators of intercellular communication that transport proteins, lipids, messenger RNAs, and non-coding RNAs between cells and organs. EVs released from adipose tissue, placenta, immune cells, endothelial cells, and other tissues play important roles in regulating inflammation, metabolism, vascular function, and tissue remodeling [50–52]. Obesity is associated with alterations in EVs abundance and cargo composition. Adipose tissue-derived EVs from obese individuals contain inflammatory cytokines, lipids, and microRNAs that can influence insulin signaling, macrophage activation, endothelial function, and cardiovascular homeostasis [50, 53]. During pregnancy, the placenta is a major source of circulating EVs, and maternal obesity alters placental EVs release and cargo, including microRNAs and inflammatory mediators that may influence fetal development and induce metabolic programming [54, 55]. These findings suggest that EVs may serve as important mediators of communication between maternal tissues, the placenta, and the developing fetus.

Although the role of EVs in parental obesity-induced developmental programming remains incompletely understood, emerging evidence suggests that obesity-associated EVs may contribute to offspring cardiometabolic risk by promoting inflammation, endothelial dysfunction, insulin resistance, and altered organ development. Potential target organs include the hypothalamus, cardiovascular system, adipose tissue, and kidneys, all of which play key roles in the regulation of energy balance and blood pressure. Furthermore, EVs-associated microRNAs, long non-coding RNAs, and proteins may represent novel biomarkers of disease risk and potential therapeutic targets. Future studies are needed to identify specific EV populations, cellular origins, and molecular cargo responsible for transmitting obesity-associated signals from parents to offspring and to determine their contribution to cardiovascular and renal dysfunction later in life.

## Increased Susceptibility to Worse Outcomes after Acute Cardiorenal Injury in Offspring of Obese Parents

Offspring from obese parents are at an increased risk of developing worse outcomes after acute injuries such as myocardial infarction (MI) and acute kidney injury (AKI) (Fig. 1). This heightened susceptibility may result from multiple factors, including epigenetic alterations, chronic low-grade inflammation, oxidative stress, endothelial dysfunction, and pre-existing cardiovascular and renal abnormalities that increase vulnerability to tissue injury.

### Offspring from Obese Parents Exhibit Greater Mortality Following Myocardial Infarction (MI)

We demonstrated that male and female offspring from obese parents exhibit diastolic dysfunction in adulthood and experience reduced survival and worse systolic dysfunction following MI, even when maintained on a normal diet, compared to offspring from lean parents [56]. Notably, parental obesity was associated with reduced left ventricular expression of connexin 43 (Cx43), a key protein for cardiac myocyte electrical impulse propagation, in male but not in female offspring [56]. These findings suggest that parental obesity worsens post-MI cardiac outcomes and increases mortality in male as well as in female offspring, although there may be sex-specific differences in the underlying mechanisms. Dysregulated mitochondrial function and reduced nitric oxide (NO) bioavailability may impair post-MI angiogenesis and increase tissue injury and dysfunction [57, 58]. In addition, elevated pro-inflammatory cytokines (IL-6, TNF-α) and increased cardiac fibroblast activation may exacerbate post-MI remodeling, leading to worse outcomes post-injury [59, 60]. Collectively, these observations highlight the need for further investigation into the mechanisms linking mitochondrial dysfunction and inflammation to increased vulnerability to cardiac injury. Additional studies are also warranted to determine how these processes contribute to the heightened susceptibility to adverse post-MI outcomes observed in offspring from obese parents.

### Increased Renal Susceptibility to Acute Kidney Injury and Worsened Renal Function Recovery

Parental obesity predisposes their offspring to kidney dysfunction and increased susceptibility to ischemia–reperfusion injury (i.e., AKI) [18]. Male offspring from obese parents exhibited increased mortality rates and worse kidney injury scores after AKI, whereas female offspring appeared to be protected [18]. These effects are observed regardless of post-weaning diet, suggesting that parental obesity programs kidney vulnerability in a sex-dependent manner even in lean offspring, although placing the offspring in the same obesogenic diet of their parents exacerbates the vulnerability [18].

Offspring from obese parents may exhibit reduced renal resilience following injury, resulting in more severe AKI and an increased risk of CKD progression. Although the underlying mechanisms remain incompletely understood, increased renal inflammation, excessive P2X7R activation, oxidative stress, endothelial dysfunction, and impaired repair processes may contribute to worsened tubular injury and fibrosis. Furthermore, alterations in renal hemodynamics and vascular function may delay recovery and promote the progression of CKD. Future studies are needed to determine the mechanisms responsible for the heightened susceptibility to kidney injury in offspring from obese parents and to identify potential therapeutic targets.

## Impact of Maternal Versus Paternal Obesity on Offspring Cardiorenal Health

### Maternal Obesity and Cardiorenal Health

Although maternal obesity appears to be a critical factor in programming offspring for cardiorenal and metabolic abnormalities, paternal obesity also plays an important role that should not be ignored. While maternal obesity influences offspring outcomes through in utero exposure to metabolic disturbances, placental dysfunction, maternal inflammation, and epigenetic programming of oocytes, paternal obesity can induce epigenetic modifications in sperm, altering gene expression patterns that persist in the offspring into adulthood [9, 61].

Recent findings suggest that both maternal and paternal obesity contribute to offspring susceptibility to worse outcomes after acute cardiorenal injury [18, 56, 62], potentially through distinct but complementary mechanisms. Additional studies are needed to explore the specific epigenetic, inflammatory, and metabolic pathways linking paternal obesity to offspring cardiovascular and renal dysfunction, as well as potential sex-specific effects.

Maternal obesity has emerged as a major determinant of offspring cardiorenal health partly because it directly influences the intrauterine environment during critical periods of fetal development. Exposure to maternal obesity is associated with alterations in nutrient availability, hormonal signaling, inflammatory status, and placental function, all of which can affect the development of cardiovascular, metabolic, and renal regulatory systems in the offspring. Consequently, evidence from human and experimental animal studies indicates that maternal obesity increases the risk of hypertension, cardiovascular dysfunction, and renal disease in offspring, even in the absence of postnatal obesity or consumption of a high-fat diet [14, 17, 54, 61].

One of the most compelling pieces of evidence supporting the developmental origins of cardiovascular health and disease in offspring from obese mothers comes from studies in women who were obese during their first pregnancy and then lost weight through bariatric surgery before their second pregnancy [63, 64]. These studies demonstrated that siblings born during the first pregnancy exhibited elevated cardiovascular risks at adulthood, as evidenced by upregulation of glucoregulatory, inflammatory and vascular disease genes [63–65], worse lipid profile, and increased C-reactive protein (CRP) levels compared to siblings born after bariatric surgery and weight loss [65]. These findings suggest that maternal weight loss prior to pregnancy can improve offspring cardiovascular health, although the relative contributions of improvements in the intrauterine environment, oocyte epigenetic programming, and other maternal factors remain unclear.

While there is substantial evidence linking maternal obesity to increased risk of diabetes, obesity, hypertension and CVD, as well as premature death in adult offspring, its impact on the risk of CKD in offspring is less clear. Previous studies indicate that maternal obesity heightens the risk of congenital urogenital malformations, such as obstructive kidney injury, in offspring [66–68]. Importantly, this effect appears to be independent of the presence of gestational diabetes [68].

Although bariatric surgery is associated with reduced mortality in pre-dialysis patients irrespective of progression to end-stage of renal diseases (ESRD) [69], to our knowledge no studies have investigated its impact on offspring born before versus after bariatric surgery being performed in their mothers. This gap in knowledge warrants future investigations to better understand how maternal weight loss, achieved by bariatric surgery, pharmacological therapy, or lifestyle modifications, may improve renal health in offspring of formerly obese mothers.

Experimental studies using sheep, rodents and non-human primates demonstrated cardiac structural alterations in offspring from obese mothers during prenatal [19, 32, 70, 71] and postnatal life [19], as well as hypertrophy in adulthood. In late gestation, fetal hearts from obese baboons exhibited increased cardiomyocyte proliferation and myocardial fibrosis [72, 73], indicative of pathological hypertrophy. Similarly, in fetal sheep from obese mothers, increased left ventricle weight and wall thickness, larger cardiomyocyte cross-sectional area, activation of hypertrophic signaling pathways, and cardiac fibrosis were observed [72]. In fetal mice exposed to maternal obesity, systolic and diastolic dysfunction were evident, characterized by lower ejection fraction and fractional shortening, prolonged isovolumetric contraction and relaxation times, and reduced early-to-late atrial (E:A) ventricular filling ratio [74]. These findings highlight the importance of maternal obesity in programming CVD in offspring during fetal development.

Previous studies also indicate an association between maternal obesity and adverse renal effects in offspring in humans; however, direct causality has yet to be demonstrated [17, 75]. The effects of maternal obesity on offspring renal health may occur indirectly through alterations in blood glucose regulation, blood pressure, or postnatal insults [9]. Epigenetic mechanisms are also implicated in the development of CKD [76], as supported by evidence showing that renal tubules from patients with CKD exhibit significant DNA methylation changes, particularly at enhancer regions associated with increased expression of key fibrotic genes [46]. However, there are no studies, to our knowledge, that have investigated the impact of maternal obesity on epigenetic changes linked to CKD development in offspring.

### Paternal Obesity and Offspring Cardiorenal Health

Although maternal obesity has historically received greater attention, accumulating evidence indicates that paternal obesity also contributes to offspring cardiovascular, metabolic, and renal dysfunction. Experimental studies demonstrated that paternal obesity can adversely affect offspring health independent of maternal obesity, suggesting that factors transmitted through the paternal germline contribute to developmental programming. For example, obese male mice exhibit increased sperm oxidative stress, DNA damage, altered DNA methylation, and dysregulated small non-coding RNAs, changes that have been linked to impaired embryonic development and transmission of metabolic dysfunction to offspring [48, 62].

Previous studies showed that paternal obesity predisposes offspring to obesity, insulin resistance, impaired glucose tolerance, and altered lipid metabolism. For instance, Ng and colleagues [77] demonstrated that female offspring of obese male rats exhibited impaired glucose tolerance and reduced pancreatic β-cell function despite normal maternal metabolic status. Similarly, Fullston and colleagues [62] reported that paternal obesity impaired metabolic health across two generations, supporting a role for germline-mediated transmission of disease susceptibility.

Evidence also suggests adverse cardiovascular consequences in offspring of obese fathers. Previous studies demonstrated that paternal obesity induced cardiac remodeling and impaired cardiovascular function in offspring [78], and alterations in cardiac gene expression and metabolic pathways associated with increased cardiovascular risk. These findings suggest that paternal obesity can influence cardiovascular development and function independently of maternal obesity. However, the mechanisms underlying these effects remain incompletely understood but are thought to involve epigenetic alterations in sperm, including changes in DNA methylation, histone modifications, and small non-coding RNAs [48, 62]. These modifications may alter early embryonic development, placental function, and organogenesis, ultimately leading to long-term changes in cardiovascular and metabolic physiology. Although evidence for direct effects of paternal obesity on renal development and function is more limited, the metabolic, inflammatory, and cardiovascular abnormalities observed in offspring may increase susceptibility to renal dysfunction and CKD later in life. Additional studies are needed to define the contribution of paternal obesity to offspring cardiorenal disease and to identify the molecular pathways involved.

## Potential Interventions to Reduce Offspring Cardiorenal Risk

Because parental obesity is increasingly recognized as a major contributor to offspring cardiorenal dysfunction, interventions aimed at improving parental health before conception and during pregnancy represent promising strategies for reducing disease risk in subsequent generations. Studies in humans and experimental models suggest that weight loss, improved nutrition, and increased physical activity before pregnancy can improve metabolic health and may attenuate adverse developmental programming in offspring [79–82]. One of the strongest pieces of evidence supporting the benefits of preconception intervention comes from studies of women who underwent bariatric surgery prior to pregnancy. Offspring conceived after maternal weight loss exhibited improved cardiometabolic profiles compared with siblings born before surgery, suggesting that improving maternal health before conception may reduce offspring disease risk [63, 65]. Similarly, experimental studies have demonstrated that maternal exercise before and during pregnancy improves metabolic function, reduces inflammation, and enhances cardiovascular health in offspring [83–85]. Although less extensively studied, emerging evidence suggests that paternal interventions may also influence offspring health. Improvements in paternal diet, body weight, and metabolic status have been shown to modify sperm epigenetic profiles and improve metabolic outcomes in offspring in animal models [48, 62]. These findings support the concept that both maternal and paternal health before conception contribute to offspring disease susceptibility.

Despite these promising observations, important questions remain regarding the timing, duration, and type of intervention required to maximize offspring health benefits. Future studies are warranted to determine whether lifestyle interventions, pharmacological therapies, or bariatric procedures implemented before conception or during pregnancy can prevent the development of cardiovascular and renal dysfunction in offspring and improve offspring resilience to cardiorenal injury later in life.

## Perspectives and Future Directions

The adverse impact of parental obesity on their offspring's metabolic and cardiorenal health later in life is a growing concern, particularly as obesity rates continue to rise globally. Epidemiological and experimental evidence strongly supports the concept that offspring born to obese parents are at a significantly higher risk of developing metabolic dysfunction, hypertension, CVD, and renal disease. These detrimental outcomes may arise due to a combination of genetic, epigenetic, intrauterine, and environmental factors that lead to overactivation of the sympathetic nervous system, dysregulated leptin-melanocortin signaling, and heightened inflammation through pathways such as P2X7R activation. In addition, epigenetic programming of gametes of the parents as well as early-life programming via mechanisms such as alterations in DNA methylation and extracellular vesicles may contribute to long-term health risks.

Understanding these pathways is crucial for developing interventions that may mitigate the risks of cardiorenal diseases in future generations. Strategies focusing on preconception and antenatal lifestyle modifications as well as pharmacotherapy and metabolic surgery could potentially prevent or reduce the severity of these adverse outcomes, emphasizing the importance of parental health before and during pregnancy. The sex-specific differences observed in experimental models of parental obesity further highlight the complexity of this issue and the need for targeted approaches for parents as well as their offspring. Additional studies are essential to unravel the mechanisms contributing to offspring cardiorenal dysfunction and to develop preventive measures for this emerging public health challenge.

## Key References

**do Carmo JM, Omoto ACM, Hall JE, Dai X, Ladnier EC, Mouro MC, Tosta OES, Wang Z, Li X, and da Silva AA. Parental obesity predisposes male and female offspring to exacerbated cardiac dysfunction and increased mortality after myocardial infarction. *Am J Physiol Heart Circ Physiol*. 2025;328:H1039–H1050.
  - **Importance:** This study demonstrates that parental obesity not only impairs baseline cardiac function but also markedly increases susceptibility to myocardial infarction, resulting in greater cardiac dysfunction and mortality in adult offspring. These findings establish parental obesity as a determinant of cardiovascular resilience later in life.
**do Carmo JM, Hall JE, Dai X, Aitkens N, Larson K, Luna-Suarez EM, Wang Z, Omoto ACM, Mouton A, Li X, Furukawa LNS, Woronik V, and da Silva AA. Parental obesity predisposes offspring to kidney dysfunction and increased susceptibility to ischemia-reperfusion injury in a sex-dependent manner. *Am J Physiol Renal Physiol*. 2024;326: F727–F736.
  - **Importance:** This work provides novel evidence that parental obesity programs long-term renal dysfunction and increases susceptibility to acute kidney injury in offspring through sex-dependent mechanisms, expanding the understanding of developmental programming beyond metabolic disease.
**do Carmo JM, Dai X, Aitken N, Larson KM, Omoto ACM, Gulke RR, Wang Z, Li X, Mouton AJ, Hall JE, and da Silva AA. Sex differences in weight gain, blood pressure control, and responses to melanocortin-4 receptor antagonism in offspring from lean and obese parents. *Am J Physiol Regul Integr Comp Physiol*. 2023;325: R401–R410.
  - **Importance:** This study identifies sex-specific alterations in melanocortin signaling and blood pressure regulation in offspring of obese parents, providing mechanistic insight into neurohumoral pathways contributing to obesity-associated hypertension.
**da Silva AA, Moak SP, Dai X, Borges GC, Omoto ACM, Wang Z, Li X, Mouton AJ, Hall JE, and do Carmo JM. Parental obesity alters offspring blood pressure regulation and cardiovascular responses to stress: role of P2X7R and sex differences. *Am J Physiol Regul Integr Comp Physiol*. 2022;322:R421–R433.
  - **Importance:** This study identifies activation of P2X7 receptor signaling as a novel mechanism contributing to impaired cardiovascular regulation and exaggerated stress responses in offspring exposed to parental obesity.
**Wells JCK, Desoye G, and Leon DA. Reconsidering the developmental origins of adult disease paradigm: The 'metabolic coordination of childbirth' hypothesis. *Evol Med Public Health*. 2024;12: 50–66.
  - **Importance: **This study presents an updated conceptual framework for the Developmental Origins of Health and Disease (DOHaD), offering new perspectives on how parental metabolic status shapes long-term offspring health.
*Jahan-Mihan A, Leftwich J, Berg K, Labyak C, Nodarse RR, Allen S, and Griggs J. The Impact of Parental Preconception Nutrition, Body Weight, and Exercise Habits on Offspring Health Outcomes: *A Narrative Review. Nutrients*. 2024;16.
  - **Importance:** This comprehensive review summarizes current evidence on how both maternal and paternal preconception health influence offspring cardiometabolic outcomes and discusses opportunities for preventive interventions.
*Liu W, Ren L, Fang F, and Chen R. Maternal pre-pregnancy overweight or obesity and risk of birth defects in offspring: Population-based cohort study. *Acta Obstet Gynecol Scand*. 2024;103: 862–872.
  - **Importance:** This large population-based study provides strong clinical evidence linking maternal obesity before pregnancy with an increased risk of congenital anomalies, emphasizing the importance of preconception health.

## Data Availability

No datasets were generated or analysed during the current study.
